# Adult vasovagal syncope with abdominal pain diagnosed by head-up tilt combined with transcranial doppler: a preliminary study

**DOI:** 10.1186/s12883-024-03623-1

**Published:** 2024-04-10

**Authors:** Jingyi Wang, Hua Li, Xuming Huang, Huoyou Hu, Baorong Lian, Daxue Zhang, Jiarui Wu, Liming Cao

**Affiliations:** 1 Faculty of Chinese Medicine, Macau University of Science and Technology, Macau, China; 2 Department of Neurology, Zhuhai Hospital of Integrated Traditional Chinese and Western Medicine, Zhuhai, China; 3 Affiliated Hospital of the Faculty of Chinese Medicine, Macao University of Science and Technology, Macau, China; 4 Department of Gastroenterology, Shenzhen baoan Shiyan People’s Hospital, Shenzhen, China; 5 Department of Neurology, The First Affiliated Hospital of Shenzhen University, Shenzhen, China; 6 Shantou University Medical College, Shantou University, Shantou, China; 7 Clinical Medical College of Shenzhen Second People’s Hospital, Anhui Medical University, Hefei, China; 8 The First School of Clinical Medicine, Guangdong Medical University, Zhanjiang, China; 9 Clinical Medical College of Shenzhen Second People’s Hospital, Guangxi University of Chinese Medicine, Nanning, China; 10 Hunan Provincial Key Laboratory of the Research and Development of Novel Pharmaceutical Preparations, Changsha Medical University, Changsha, China

**Keywords:** Vasovagal syncope, Head-up tilt test, Transcranial doppler, Abdominal pain, Synchronous multimodal test

## Abstract

**Background:**

Syncope is a common condition that increases the risk of injury and reduces the quality of life. Abdominal pain as a precursor to vasovagal syncope (VVS) in adults is rarely reported and is often misdiagnosed.​.

**Methods:**

We present three adult patients with VVS and presyncopal abdominal pain diagnosed by synchronous multimodal detection (transcranial Doppler [TCD] with head-up tilt [HUT]) and discuss the relevant literature.

**Results:**

Case 1: A 52-year-old man presented with recurrent decreased consciousness preceded by six months of abdominal pain. Physical examinations were unremarkable. Dynamic electrocardiography, echocardiography, head and neck computed tomography angiography, magnetic resonance imaging (MRI), and video electroencephalogram showed no abnormalities. Case 2: A 57-year-old woman presented with recurrent syncope for 30 + years, accompanied by abdominal pain. Physical examination, electroencephalography, and MRI showed no abnormalities. Echocardiography showed large right-to-left shunts. Case 3: A 30-year-old woman presented with recurrent syncope for 10 + years, with abdominal pain as a precursor. Physical examination, laboratory analysis, head computed tomography, electrocardiography, and echocardiography showed no abnormalities. Syncope secondary to abdominal pain was reproduced during HUT. Further, HUT revealed vasovagal syncope, and synchronous TCD showed decreased cerebral blood flow; the final diagnosis was VVS in all cases.

**Conclusions:**

Abdominal pain may be a precursor of VVS in adults, and our findings enrich the clinical phenotypic spectrum of VVS. Prompt recognition of syncopal precursors is important to prevent incidents and assist in treatment decision-making. Abdominal pain in VVS may be a sign of sympathetic overdrive. Synchronous multimodal detection can help in diagnosing VVS and understanding hemodynamic mechanisms.

## Background

Syncope is a common condition that general practitioners, cardiologists, and neurologists evaluate. It is defined as a transient loss of consciousness caused by global cerebral hypoperfusion [1], characterized by rapid onset, short duration, and complete spontaneous recovery [2]. Vasovagal syncope (VVS), also known as neurally mediated syncope, is diagnosed based on a history of a specific trigger, classical symptoms, and a positive tilt-table test. VVS is the most common form of syncope, accounting for 66% of cases [3]. Syncope causes consequential economic burdens, significantly impacts the quality of life, and can result in accidental injury, particularly in older adults [4, 5]. Typical VVS can usually be diagnosed based on history and preliminary examination; however, atypical VVS can be more difficult to diagnose [6].

The lifetime prevalence of syncope is approximately 42%, with an annual incidence of 6% [7]. Syncope occurs when cerebral blood flow (CBF) falls below a critical limit. The cerebral circulation has complex, finely tuned autoregulatory mechanisms to ensure that the blood supply to the brain can meet the high metabolic demands of the neuronal tissue [8]. Cerebral autoregulation (CA) mechanisms help defend the brain against hypoperfusion when perfusion pressure falls during standing. VVS results from decreased CBF secondary to a decrease in heart rate and blood pressure (BP) that exceeds the CA function [9, 10].

Transcranial Doppler (TCD) provides real-time measurements of CBF velocity, which can be useful in syncope workup. Syncope typically occurs when patients are upright and can be preceded by presyncopal symptoms, including dizziness or light-headedness, nausea, weakness, pallor, diaphoresis, and tachypnea [11]. Patients can experience a graying of vision or muffling of sounds before losing consciousness. Furthermore, rare signs of presyncope include oral automatisms [1]. Abdominal pain and nausea may occur before or during episodes in children with VVS [12]. Prodromes are more common in patients with early-onset syncope [13]; abdominal pain as the first symptom of syncope in adults is rarely reported. Nevertheless, we present three adult patients with VVS and abdominal pain as presyncope, diagnosed using the synchronous head-up tilt test (HUT) combined with TCD, and discuss relevant literature for improving the management of atypical VVS syncope.

## Methods

**Fig. 1:**
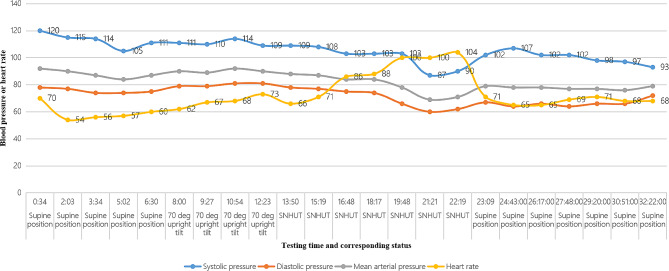
The trend-line of blood pressure and heart rate in the patient during head-up tilt test *SNHUT* sublingual nitroglycerin head-up tilt test

We present the clinical and hemodynamic features of three adult patients with VVS and abdominal pain as presyncopes, diagnosed by synchronous multimodal detection (HUT combined with TCD, Fig. 1), and discuss the relevant literature. The study was approved by the ethics review board of the First Affiliated Hospital of Shenzhen University (No. 202,304,133,010). It was performed per the ethical standards laid down in the 1964 Declaration of Helsinki and its later amendments or comparable ethical standards.

### Diagnostic criteria for VVS

The diagnostic criteria for VVS are as follows [14–16]:

1. There was a transient loss of consciousness caused by insufficient cerebral blood supply.
2. The patient fell to the ground because of the loss of muscle tension during syncope and could not maintain a normal posture. Generally, the attack occurred suddenly and resolved rapidly.
3. Accompanied by associated symptoms like sweating, a sensation of warmth, nausea, and paleness, along with the distinctive low BP and slower than normal heart rate [15].
4. Occurs with an upright posture maintained for over 30 s or due to exposure to emotional stress, pain, or medical environments.
5. A positive response to a HUT with a vasovagal reaction (hypotension and/or bradycardia) [16].

Patients with neurological disorders such as epilepsy, endocrine disorders like hypoglycemia, drug poisoning including overdose, alcohol intoxication, head injuries from trauma, and other factors leading to loss of consciousness were not included.

### Operation process of HUT [17]

1. Patients fasted for at least 4 h and were kept in a quiet test room with ambient light and temperature. Furthermore, they were placed in the supine position for at least 10 min, and baseline BP and heart rate were measured.
2. The patient remained upright (70° tilt) for 20 min and stopped at any time if syncope was induced.
3. When syncope did not occur during the classic tilt table test, the patient was administered sublingual 0.25 mg nitroglycerin in an upright position for 20 min or until syncope or presyncope occurred.

We used conventional intermittent BP measurements with an electronic blood pressure monitor, measuring BP once every minute, and utilized 12-lead electrocardiogram (ECG) monitoring to track heart rate and ECG changes. We used the HUT822-A upright tilting table (Juchi Medical Technology Co., Beijing, China).

### Operation of synchronous CBF monitoring using TCD

CBF was monitored during HUT using an ems-9 TCD (Delikai Medical Equipment Co., Shenzhen, China) with a 1.6-MHz probe during HUT. Peak systolic and diastolic blood flow, with mean CBF velocities and vascular resistance index of the middle cerebral artery, were recorded after the head frame was fixed.

### Determination of a positive result of HUT [18]

A positive HUT test was defined as either:

1. Onset of syncope or presyncope during the baseline (in the upright position) or drug-induced test, systolic BP < 80 mmHg, diastolic BP < 50 mmHg, mean arterial pressure decreased by > 25%, or systolic BP dropped below 90 mmHg, accompanied by evident presyncope or syncope;
2. Electrocardiography showed sinus bradycardia (< 40 bpm), sinus arrest for over 3 s, or heart rate decreased by > 20%; transient ≥ second-degree atrioventricular block, junctional rhythm TCD showed decreased CBF, and electroencephalography showed slow waves;
3. During carotid sinus massage, cardiac arrest > 3 s, and systolic BP decreased by > 30 mmHg after 3 min in the upright position, systolic BP decreased by ≥ 20 mmHg, and diastolic BP decreased by ≥ 10 mmHg.

### Inclusion criteria

Patients with the following criteria were included: abdominal pain prior to suspected syncope, age 18–80 years, simultaneous HUT and TCD could be completed, and ability to sign informed consent.

### Exclusion criteria

Patients with any of the following criteria were excluded: severe intracranial or extracranial vascular stenosis, severe coronary artery stenosis, severe aortic or mitral valve stenosis, severe hypertrophic obstructive cardiomyopathy, severe anemia, severe arrhythmia, moderate to severe hypertension, or pregnancy.

### Data collection

A standard approach was used to collect patient history (including chief complaint, history of present illness, past medical history, personal history, physical examination, main auxiliary examination results, diagnosis, and treatment).

The specific laboratory analysis methods are as follows: blood glucose (hexokinase method), total bilirubin (vanadate oxidation method), low-density lipoprotein cholesterol (direct method), C-reactive protein (immunoturbidimetric method), creatinine (creatinine oxidase method), homocysteine (enzyme cycling method), electrolytes (ion-selective electrode method), D-dimer (immunoturbidimetric method), thyroid function (enzyme chemiluminescence method), red blood cell count (colorimetric method), syphilis antibodies (rapid plasma reagent test), and human immunodeficiency virus antibodies (enzyme-linked immunosorbent assay).

#### ECG examination procedure

First, the patient reclined on the examination table, exposing the chest and limbs. The electrodes were attached to the chest and limbs, and the patient was asked to remain still while the doctor recorded the ECG waveform. Cardiac electrical activity was assessed based on the waveform.

#### Chest X-ray examination procedure

The patient stood before the X-ray machine in a standard position. X-ray images from the front and back were taken after the patient inhaled deeply and held their breath.

#### Carotid artery ultrasound examination procedure

An EPIQ 7 C Color Doppler Ultrasound (Philips Healthcare, Best, Netherlands) equipped with a 9-MHz probe was used. The patient lied in the supine position, exposing their neck. The carotid artery was scanned using an ultrasound probe and the artery’s condition was assessed based on the images and blood flow signals.

## Results

Three of the 60 patients with VVS were successfully recruited between January 2021 and January 2023, and the specific clinical and hemodynamic features of the selected individuals are as follows.

### Case 1

A 52-year-old man presented with recurrent transient episodes of decreased consciousness for 6 months in December 2022. The patient experienced sudden confusion with severe abdominal pain and profuse sweating while walking 6 months prior, which was relieved after 3–5 min. He experienced generalized abdominal pain (for approximately 5 min until losing consciousness) again 28 days prior, accompanied by sweating, general fatigue, and blurred vision for 3 min. Subsequently, he got up to use the toilet, followed by syncope without foaming at the mouth or convulsions, and consciousness was regained 2 min later. The patient had a history of mild fatty liver, benign prostatic hyperplasia, and cholecystic polyps; however, he had no history of infectious diseases, smoking, drinking alcohol, toxic exposure, or hereditary diseases.

Furthermore, a physical examination at admission revealed a pulse rate of 70 bpm, BP of 116/74 mmHg, and no abnormal neurological signs. The abdomen was flat and soft with normal bowel sounds, and no apparent masses.

Laboratory analysis revealed homocysteine, C-reactive protein, D-dimer, troponin, electrolytes, fasting glucose, and glycated hemoglobin levels were within normal ranges. In addition, red blood cell counts and liver, kidney, and thyroid function measurements were normal, and tests for human immunodeficiency virus and syphilis antibodies were negative. Urine analysis showed a weakly positive urine occult blood test.

**Fig. 2:**
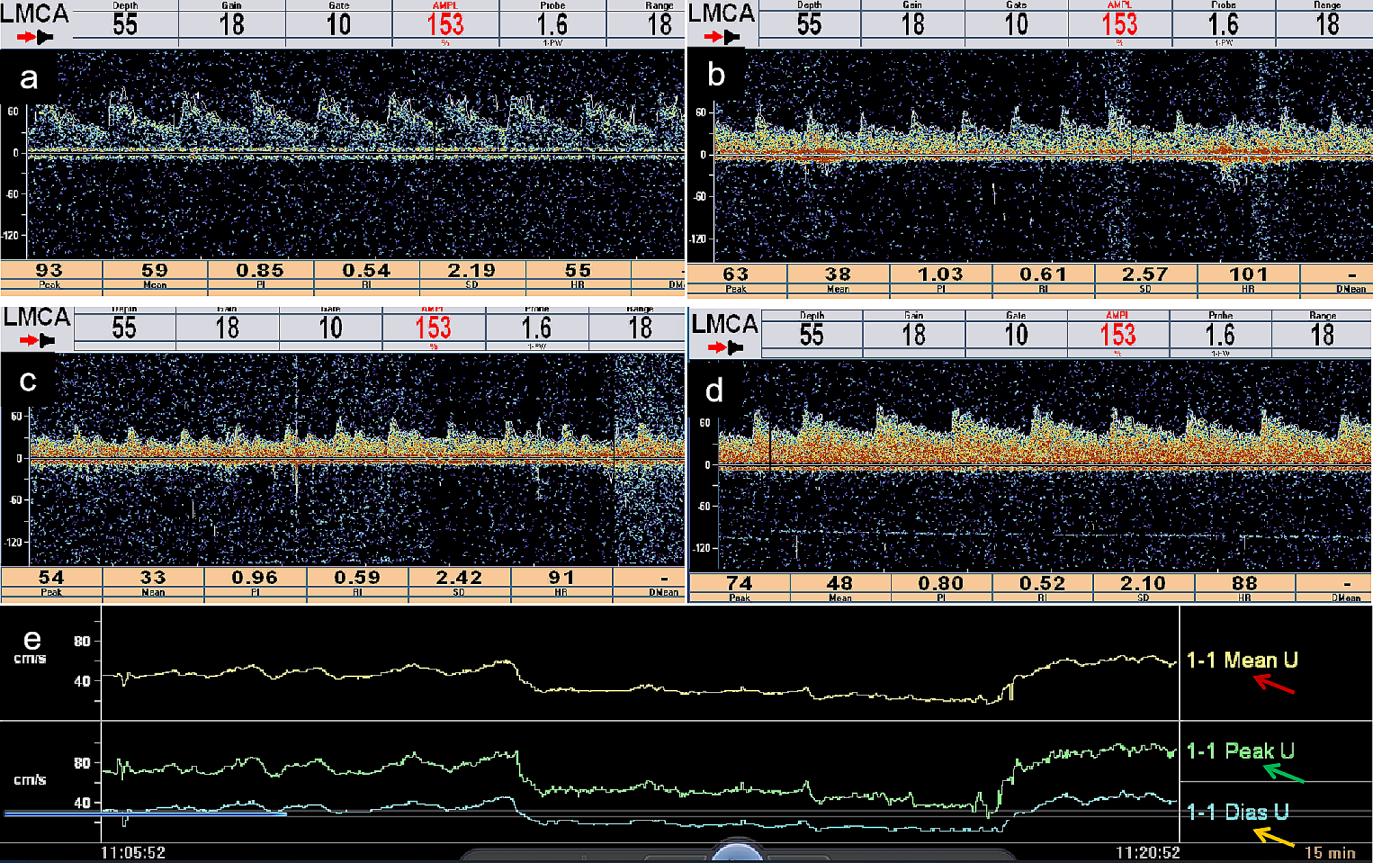
Synchronous transcranial Doppler (TCD) displays real-time cerebral blood flow (CBF) at different stages **a**. Baseline CBF in the supine position at rest **b**. TCD in the upright position shows decreased CBF with a high-resistance waveform **c**. Mean CBF velocity during the drug trials continued to decrease, and the pulsatility index increased **d**. Mean CBF velocity, pulsatility index, and waveform quickly returned to baseline levels when the participant returned to the supine position **e**. CBF velocity trend-line of mean (red arrow), peak systole (green arrow) and end diastole (yellow arrow)

Chest computed tomography (CT) revealed scattered fibrous foci in both lungs with tiny lung nodules. Electrocardiography, carotid artery ultrasound, brain MRI, and a 15-h video-electroencephalogram monitoring showed no abnormalities. In addition, 24-h dynamic electrocardiography showed an incomplete right bundle branch block, occasional multi-source premature atrial beats, occasional premature ventricular beats, and ST-t changes with increased heart rate. Echocardiography revealed decreased left ventricular diastolic function. Moreover, 24-h ambulatory BP monitoring revealed intermittent low BP. Head and neck CT angiography (CTA) revealed mild cerebral and carotid arteriosclerosis. Furthermore, HUT findings supported the diagnosis of VVS (Fig. 1), and synchronous TCD showed an obvious decrease in CBF during syncope (Fig. 2). Oxiracetam and *Ginkgo biloba* were administered to enhance brain cell metabolism and brain circulation, respectively, for 6 days. No syncope or abdominal pain occurred after admission. A final diagnosis of VVS (Type 3) was made. No VVS recurrence was noted during the 8-month follow-up post discharge.

### Case 2

A 57-year-old female was admitted to the hospital in June 2022 with recurrent episodes of unconsciousness for over 30 years. The patient had recurrent syncope with falls starting 30 years prior and was unconscious for a few seconds to a few minutes without limb convulsions or incontinence. Syncope occurred once every five years and occasionally during urination and defecation. A previous ECG revealed no abnormalities. Nevertheless, syncope recurred prior to admission, accompanied by generalized abdominal pain lasting approximately 3 min before losing consciousness, with no presence of diarrhea or chest tightness. She had no history of hereditary diseases, major trauma, smoking, drinking, or toxic exposure.

Physical examination on admission revealed BP of 106/69 mmHg, a flat, soft abdomen with normoactive bowel sounds, no masses, and no abnormal neurological signs.

Laboratory analysis revealed the following: red blood cell count, 3.79 × 10^12^/L (reference range: 1.25–3.5 × 10^12^/L); hemoglobin, 113.0 g/L (reference range: 115–150 g/L); triglycerides, 3.35 mmol/L (reference range: <1.7 mmol/L); apolipoprotein E, 60.06 mg/L (reference range: 29.0–53.0 mg/L); and thyrotropin, 0.490 µIU/mL (reference range: 0.55–4.78 µIU/mL). Red blood cell count and measures of liver, kidney, and coagulation functions were normal. Additionally, levels of serum electrolytes, fasting blood sugar, glycosylated hemoglobin, D-dimer, and troponin I were within the normal range.

ECG revealed ST-segment changes. A 24-h ambulatory electrocardiogram showed sinus rhythm with occasional atrial premature beats or bradycardia, ST-segment changes, and normal heart rate variability. Chest X-ray and ultrasound of the liver, gallbladder, pancreas, spleen, and uterine accessories were normal. Carotid artery ultrasound showed bilateral intima-media thickening with plaque formation. Head and carotid artery CTA revealed mild cerebral arteriosclerosis and contrast-enhanced echocardiography detected large right-to-left shunts. Enhanced transesophageal echocardiography revealed no patent foramen ovale and a moderate number of microvesicles were shunted from the right superior pulmonary vein into the left atrium. Pulmonary artery CTA and 15-h video electroencephalography showed no obvious abnormalities, and brain MRI showed mild leukoencephalopathy in the right frontal subcortex. HUT revealed VVS, and synchronous TCD showed an obvious decrease in CBF during syncope. Based on medical history and auxiliary examination findings, the final diagnosis was VVS (Type 3). Thereafter, the patient experienced an improvement in cerebral circulation, cholesterol reduction, and symptomatic treatment after admission and felt no further discomfort.

### Case 3

A 30-year-old female presented with more than 10 years of recurrent syncope in February 2023. The first syncopal episode was painful, secondary to hitting her elbow in elementary school. The second episode occurred at the age of 18 when the patient experienced abdominal pain for 2–3 min and then syncope. Five years prior to presentation, the patient experienced another episode of syncope during a hospital visit. One week prior, the patient experienced severe lower abdominal pain while chatting and wanted to defecate, which lasted for approximately 2 min, and was followed by syncope. One day prior, the patient experienced abdominal discomfort after standing for more than 20 min on the subway, which lasted for 1–2 min, followed by decreased consciousness with fainting and sweaty palms, which gradually improved after 2–3 min. The patient was then immediately transferred to a local hospital. Consequently, head CT, ECG, routine blood tests, and biochemical analyses were conducted; however, no obvious abnormalities were observed. Therefore, the patient’s medical history was unremarkable.

Physical examination at admission showed no obvious neurological or abdominal abnormalities. Blood analysis showed that white blood cell count, platelet count, and neutrophil percentage were normal. The liver, kidney, and coagulation functions were also normal. In addition, fasting blood glucose, blood electrolytes, and pro-brain natriuretic peptide levels were within normal limits.

Electrocardiography, 24-h dynamic electrocardiography, and echocardiography showed no obvious abnormalities. HUT showed VVS and synchronous TCD showed an obvious decrease in CBF during syncope. Interestingly, presyncope (abdominal pain) was induced during HUT testing and was immediately relieved after lying flat. The final diagnosis was VVS (Type 3) based on the medical history and auxiliary examination findings.

The summarized clinical and hemodynamic characteristics of all three cases are outlined below:

1. All three cases were diagnosed with VVS Type 3.
2. All three patients were adults with onset varying from 6 months to 30 years.
3. Primary symptoms comprised recurrent episodes of reduced consciousness or syncope.
4. Preceding the episodes, all patients encountered intense abdominal pain lasting 1–5 min.
5. Throughout the episodes, there were no limb convulsions or urinary incontinence.
6. The results of HUT in all three cases confirmed VVS, and simultaneous TCD indicated a significant decrease in CBF during syncope. Particularly in Case 3, when abdominal pain recurred during HUT, TCD displayed a noteworthy decrease in intracranial blood flow velocity.

## Discussion

**Fig. 3:**
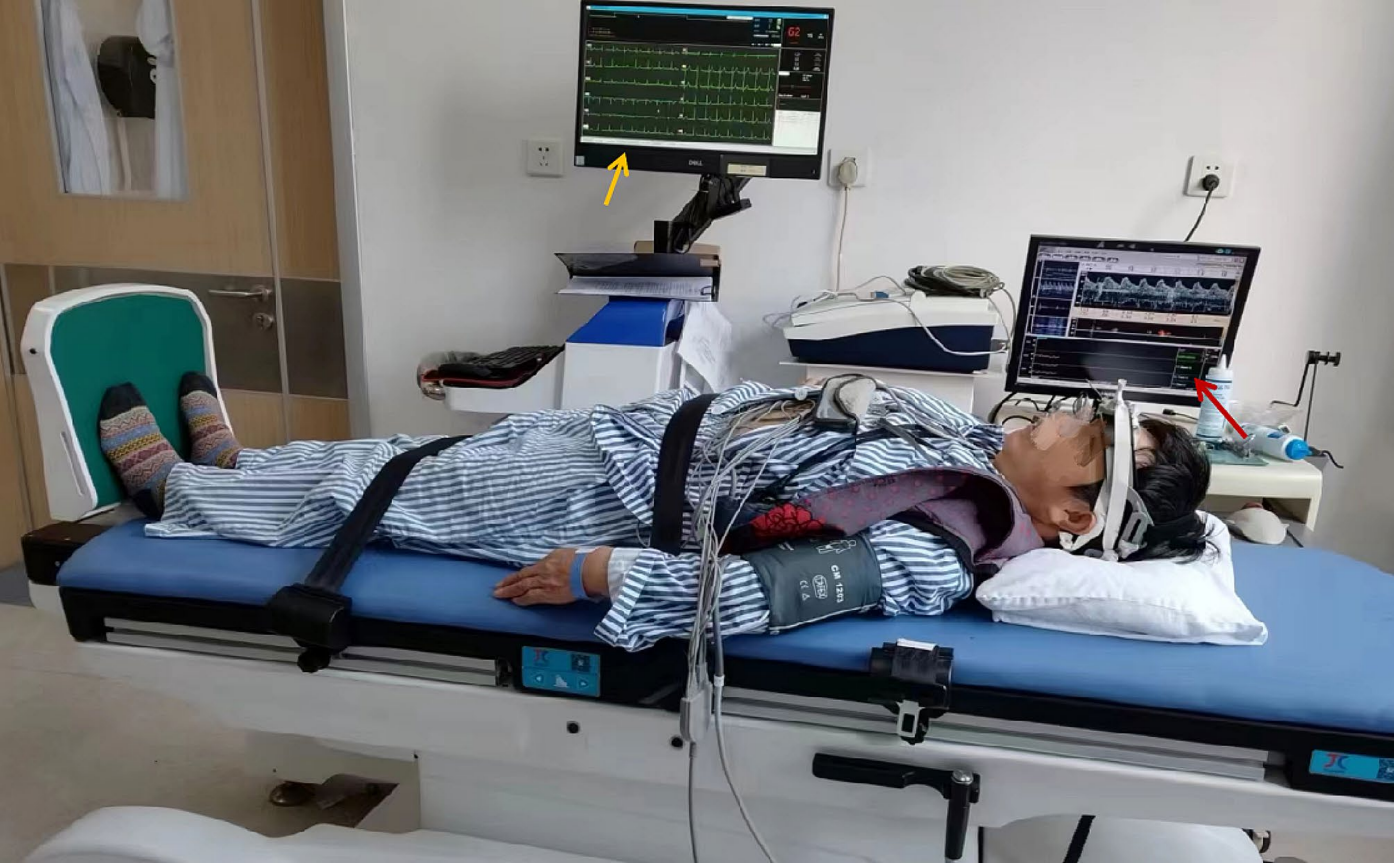
Operation of synchronous head-up tilt (HUT) test combined with transcranial Doppler (TCD) sonography The participants wore the head frame of the TCD and lay flat on an upright tilting bed. Changes in blood pressure, heart rate, and respiration in different positions and drug trials were observed on the HUT screen (yellow arrow), and the TCD screen shows synchronous cerebral blood flow status and a trend line (red arrow)

Adult VVS with abdominal pain as a precursor is rarely reported and easily misdiagnosed as abdominal epilepsy or acute abdomen. Timely diagnosis of VVS, with abdominal pain as a precursor, is crucial for decision-making. Moreover, abdominal pain in VVS may indicate sympathetic overdrive or cerebral circulatory insufficiency stimulating the visceral sensory system. HUT combined with TCD (Fig. 3) not only has advantages in diagnosing VVS but can also reveal real-time cardiocerebral hemodynamic changes and aid in understanding the pathogenesis of syncope and presyncope.

### Clinical characteristics of abdominal syncope

Three patients had atypical VVS with celialgia as a precursor, and abdominal pain was relieved after the syncope attack; celialgia could not be explained by gastrointestinal diseases, and this kind of VVS is called “abdominal syncope.” The core manifestations of abdominal syncope are sudden mild-to-severe abdominal pain lasting for several minutes, followed by syncope with or without other presyncope symptoms. There are some reports of celialgia during presyncope in children but rarely in adults. For instance, 33 (35.5%) children with VVS had gastrointestinal discomfort (celialgia, nausea, and vomiting accounting for 16.1%, 21.5%, and 12.9%, respectively) before the syncope attack, and 27 (40.9%) had gastrointestinal manifestations (celialgia accounted for 9.1%) during HUT [12]. Our findings suggest that abdominal pain is not as rare as previously thought as a presyncope symptom in adults; however, it is a non-specific symptom that clinicians do not assess or is misjudged as other diseases. During multimodal detection, we reproduced abdominal pain as a symptom of presyncope, which had a close temporal correlation with CBF changes. Therefore, we concluded that abdominal pain is a precursor of VVS. The onset of abdominal pain is of short duration, usually alleviated with the return of consciousness, and can be accompanied by cold sweating, facial pallor, and other symptoms of sympathetic excitation.

### Pathophysiological mechanism of abdominal syncope

Syncope is a common manifestation of several disorders with diverse mechanisms. Patients with VVS present with autonomic nervous system dysfunction during HUT. Studies on heart rate variability in children confirmed the dysregulation of the autonomic nervous system in children with VVS [19]. Functional abdominal pain is associated with generalized autonomic nervous system dysfunction [20]. The known function of the sympathetic nervous system as the motility “brake” suggests that pain could manifest unmodulated peristalsis, resulting in abdominal cramps [20]. During a syncopal episode, hypotension causes sympathetic excitation and secondary spasmodic contraction of systemic blood vessels (e.g., veins of viscera, skin) to increase returned blood volume to the heart. Specifically, skin vasoconstriction manifests as pallor and cold sweating, and gastrointestinal contractions manifest as abdominal pain. Functional abdominal pain has been linked to autonomic dysfunction [21, 22]. CA refers to the intracranial arterioles that act through contraction or diastole to maintain relatively stable CBF during systemic BP changes. Several paradigms to study dynamic CA have been developed by measuring CBF velocity with TCD in response to BP changes [23]. The autonomic nervous system mediates the contraction and relaxation of cerebral arterioles; therefore, CA is related to autonomic nervous function. Furthermore, the onset of VVS is caused by cerebral hypoperfusion secondary to dysregulation of the CA. Our patients, diagnosed with abdominal pain accompanied by decreased CBF during multimodal testing, were subsequently relieved when the CBF returned to normal. Another proposed mechanism is that decreased CBF may affect the blood supply to the visceral sensory systems in the brain, stimulating abdominal discomfort.

### Diagnosis and differential diagnosis of abdominal syncope

Syncope is differentiated from various causes of altered consciousness, such as seizures, metabolic disturbances, and psychiatric events. In VVS, amnesia due to loss of consciousness is common and can often lead to misdiagnosis [6]. Abdominal pain of unknown origin is rarely considered to be associated with syncope. Syncope preceded by abdominal pain is easily misdiagnosed as abdominal epilepsy [24], acute abdomen (such as renal artery aneurysm rupture) [25], nutcracker syndrome [26], and other conditions. In Cases 1 and 2, we took a thorough history and review of systems in these patients to rule out gastrointestinal, renal, gynecological, or other intra-abdominal issues that could potentially contribute. Gastrointestinal symptoms such as abdominal pain have significant implications in diagnosing VVS in children [27]; however, the warning role of abdominal pain in VVS is not yet emphasized in adults.

With severe cerebral hypoperfusion, brief convulsions of the limbs may be observed. A previous study has shown that 5.75% of patients with VVS may be misdiagnosed as having epilepsy because myoclonic jerky movements are observed during syncope [28]. HUT, a recognized stimulus to VVS [29], has significant clinical value in the differential diagnosis of VVS [30]. Furthermore, the primary and immediate cause of syncope is cerebral hypoperfusion. Thus, we used HUT combined with TCD to reproduce syncope episodes and observed changes in cardiocerebral hemodynamics and corresponding symptoms, indicating that this multimodal test has important value in the diagnosis and differential diagnosis of VVS.

### The value of HUT combined with TCD for the diagnosis of VVS

The HUT is a standard method for evaluating unexplained syncope. Standing causes a gravitational shift of blood into the venous capacitance system of the legs and pelvis, and prolonged standing causes fluid movement into interstitial spaces and a lack of muscular pumping, both resulting in reduced venous return. In healthy individuals, compensatory mechanisms cause an increase in vascular resistance and heart rate to help maintain adequate cerebral perfusion [11]. Furthermore, the HUT can provide similar but more controlled orthostatic stress to evaluate susceptibility to vasovagal reactions. The HUT can only detect changes in respiration, heart rate, and BP; however, HUT combined with TCD can monitor real-time changes in CBF to remedy the deficiency in content and timeliness during HUT alone. Therefore, we believe that multimodal examination is a superior method of diagnosing VVS. Our experience with multimodal tests has shown that changes in CBF velocity and the index of vascular resistance often precede changes in BP; thus, operators can predict syncope attacks in advance to facilitate the adoption of safety measures.

### Treatment

The treatment of VVS with abdominal pain requires a comprehensive approach that considers both non-pharmacological and pharmacological interventions. Non-pharmacological treatments such as physical counterpressure maneuvers have been shown to be effective and cost-efficient in managing VVS, especially in patients with recognizable prodromal symptoms [31]. Additionally, the use of progressively prolonged periods of enforced upright posture, known as “tilt training,” may reduce syncope recurrence in highly motivated patients with recurrent vasovagal symptoms [32]. Other therapies for VVS include increased salt and water intake and various drug treatments (such as atenolol [33] and midodrine [34]). Furthermore, the effectiveness of β-blockers in preventing VVS has been found to be age-dependent, with potential effectiveness in older patients [35]. However, most drugs are still under investigation [36].

### Limitations

It is important to note that drawing definitive conclusions based on a small group of three patients may not be statistically robust or generalizable to a larger population. While our study has provided insights into the clinical characteristics and potential mechanisms of abdominal pain associated with VVS, further research involving larger sample sizes is necessary to validate these findings and establish more conclusive evidence. Additionally, we did not utilize beat-to-beat BP monitoring, which is a technical limitation, but we are confident that this does not impact the overall findings.

## Conclusion

Timely identification and accurate diagnosis of VVS-related abdominal pain remain crucial for effective treatment decisions and avoiding misdiagnosis. The hypothesis that VVS-related abdominal pain may be linked to sympathetic excitation or inadequate CBF stimulation of visceral sensory neurons warrants further investigation through comprehensive multicenter studies with larger cohorts. The combination of HUT testing with TCD for diagnosing VVS is promising.

## Data Availability

All data generated or analyzed during this study are included in this published article.

## Abbreviations

CA: cerebral autoregulation
CBF: cerebral blood flow
CT: computed tomography
CTA: computed tomography angiography
HUT: head-up tilt
MRI: magnetic resonance imaging
TCD: transcranial Doppler
VVS: vasovagal syncope
